# Effects of Electroacupuncture on the Gut Microbiome in Cisplatin-Induced Premature Ovarian Failure Mice

**DOI:** 10.1155/2022/9352833

**Published:** 2022-03-14

**Authors:** Qi-da He, Jing-jing Guo, Qi Zhang, Yuen-ming Yau, Yue Yu, Zheng-hong Zhong, Zi-yan Tong, Zong-bao Yang, Min Chen

**Affiliations:** ^1^Faculty of Chinese Medicine, Macau University of Science and Technology, Macau 999078, China; ^2^State Key Laboratory of Quality Research in Chinese Medicines, Macau University of Science and Technology, Macau 999078, China; ^3^Department of Traditional Chinese Medicine, Xiamen University, Xiamen 361000, China

## Abstract

Growing evidence showed that the gut microbiota was associated with premature ovarian failure (POF). Many clinical types of research had shown that electroacupuncture was effective in the treatment of POF. However, there was little research on regulating the gut microbiome of POF mice by electroacupuncture. Therefore, this study attempted to verify whether electroacupuncture could regulate the gut microbiome in POF mice. POF mice were established by being injected intraperitoneally with cisplatin (2 mg/kg) for 2 weeks. Guanyuan (CV4) and Sanyinjiao (SP6) were selected in the electroacupuncture-at-the-acupoints group (EA group). Nonacupoints around CV4 and SP6 were selected in the electroacupuncture-at-the-nonacupoints group (EN group). The EA group and EN group were treated for 3 weeks. The ovarian function was evaluated by histopathological and molecular assays. Meanwhile, the gut microbiome of all mice was detected by 16S rDNA sequencing. The results showed that EA could restore the estrous cycle and reduce the number of atresia follicles in POF mice. The levels of serum follicle-stimulating hormone and luteinizing hormone were decreased by EA. As well, the levels of serum estradiol, anti-Mullerian hormone, and *β*-glucuronidase were increased by EA. The relative expressions of PI3K, AKT, and mTOR were increased to promote the proliferation of ovarian cells in the EA group. According to the results of 16S rDNA sequencing, the abundance and diversity of the gut microbiome could be regulated by EA. The relative abundance of beneficial bacteria was increased by EA. The KEGG pathway analysis showed that the gut microbiome associated with the estrogen signaling pathway, oocyte maturation, and PI3K-AKT signaling pathway was regulated by EA.

## 1. Introduction

Nowadays, more and more patients are diagnosed with premature ovarian failure (POF) in clinics. The epidemiology shows that the incidence rate of women with POF in childbearing age is over 4% [1]. POF is the premature decline or failure of ovarian function before 40 years old. The level of serum follicle-stimulating hormone (FSH) is increased and estradiol (E_2_) is decreased in patients with POF [2]. POF can be caused by metabolic disorders, gonadotropin dysfunction, immune damage, chemical damage, genetic factors, and others. However, the aetiology remains unclear in nearly half of the patients with POF [3]. Hormone therapy (HT) can supplement exogenous hormones and relieve the symptoms caused by hormone deficiency in time [4]. However, the risk of breast cancer may be increased by long-term use of HT [5]. Therefore, an alternative or complementary therapy is needed for POF.

Electroacupuncture is one of the most popular therapies in Chinese traditional medicine. Several clinical randomized controlled trials had shown that electroacupuncture could improve ovarian function and increase the pregnancy rate in patients with POF [6].

Recently, the gut microbiome has been proved to participate in the enterohepatic circulation of estrogen to regulate the level of estrogen in serum [7]. As reported, the level of estrogen could induce a positive and negative feedback mechanism through the hypothalamus pituitary ovary (HPO) axis [8]. Although mainly estrogen is secreted from the ovary, the level of estrogen in serum may also affect the functions of the ovary [9]. Therefore, the gut microbiome may be closely related to ovarian function. However, to the best of our knowledge, there are few studies on the correlation between the gut microbiome and electroacupuncture for POF.

In this study, 16S rDNA sequencing assay was applied to investigate the effect of electroacupuncture on the gut microbiome in mice with POF. In addition, histopathological and molecular biological assays were used to evaluate the effect of electroacupuncture on ovarian function.

## 2. Materials and Methods

### 2.1. Animals

All animals were raised in the Experimental Animal Centre of Xiamen University. The study was approved by the Animal Care and Use Committee of Xiamen University (Permit Number: SCXK: 2018-0003). The experimental procedures were carried out by following per the ethical guidelines of the International Council for Laboratory Animal Science (ICLAS).

40 ICR female mice (30 g ± 5 g) were randomly divided into 4 groups (*n* = 10 each group): control group, POF group, electroacupuncture-at-the-acupoints group (EA group), and electroacupuncture-at-the-nonacupoints group (EN group). The POF mice were established by intraperitoneal injection of cisplatin (2 mg/kg) for 2 weeks in all groups except for the control group [10].

### 2.2. Electroacupuncture Treatment

According to the “Experimental Acupuncturology”, Guanyuan (CV4), Sanyinjiao (SP6), and nonacupoints (5 mm horizontally beside CV4 and 3 mm vertically above SP6) were selected. The nonacupoints did not belong to any meridians. Subsequently, all acupoints and nonacupoints were disinfected with 75% alcohol before electroacupuncture. The 0.25 mm × 13 mm acupuncture needles (Suzhou Medical Appliance Factory, Jiangsu, China) were selected. The depth of acupuncture was about 3–5 mm. The current output mode of the electroacupuncture instrument (Model SDZ-II; Suzhou Medical Appliance Factory, Jiangsu, China) was alternate of sparse wave and dense wave (intermittent wave: 4 Hz; irregular wave: 50 Hz). The POF mice in the EA group and EN group were treated by electroacupuncture 30 min daily for 3 weeks (Figure S1(a) and S1(b)).

Afterwards, all mice were anaesthetized by inhaling isoflurane at the end of electroacupuncture treatment.

### 2.3. Observation of Estrous Cycle

20 *μ*L of 0.9% NaCl solution was infused into the vagina of mice by the pipette gun and pumped gently several times. Then, the solution was sucked out onto the glass slide and stained. Vaginal exfoliated cells were observed under the light microscope. The above steps were repeated daily during the period of the experiment.

### 2.4. Histopathology Examination

Ovaries were collected and dehydrated in 10% paraformaldehyde for 48 hours after mice were sacrificed and embedded with paraffin after ethanol dehydration. Subsequently, ovaries were sliced to a thickness of 5 mm, and the morphology of the ovary was observed under the light microscope after being stained.

### 2.5. Enzyme Linked Immunosorbent Assay (ELISA)

Blood samples were collected via enucleated eyes. Blood samples were centrifuged (10000 rpm, 10 min, 4°C) to obtain the serum, and the FSH, E_2_, luteinizing hormone (LH), anti-Mullerian hormone (AMH), and *β*-glucuronidase were detected by using the ELISA kit. All experimental processes were performed according to the protocol provided by the manufacturer.

### 2.6. Quantitative Real-Time PCR (qPCR)

The relative expressions of PI3K, AKT, and mTOR in the ovary were detected by qPCR. Then, the ovary was put into phosphate buffer solution (PBS) and homogenized on ice. The homogenate was centrifuged (3000 r/min, 15 min, 4°C), and the upper liquid was collected. Afterwards, total RNA was extracted by the method of TriPure and reverse transcribed into cDNA. The CT value was derived, and the relative expressions of PI3K, AKT, and mTOR were calculated according to the 2^−△△^CT value after amplification.

### 2.7. Detection and Analysis of Gut Microbiome

Faecal samples were collected in the sterile laboratory before being sacrificed. The faecal samples were placed into sterile centrifuge tubes with PBS and mixed. All samples were centrifuged (4000 r/min, 4°C, 30 min), and precipitates were retained. After, total DNA was extracted according to the protocol of the kit (DNAzol, Thermo Fisher, USA). The DNA of bacillus coli was applied as a template for amplification. Clone databases of the 16S rDNA genes were constructed according to the DNA Sample Prep Kit (TruSeq™, Illumina, USA), and DNA sequences were detected (MiSeq, Illumina, USA). Finally, original sequences were spliced and filtered.

Principal component analysis (PCA) was applied to compare the bacterial diversity of the gut microbiome. The analysis of similarities (ANOSIM) was applied to analyze whether there was comparability between each group. In addition, the linear discriminant analysis (LDA) was applied to screen out the different species through LEfSe. Finally, the function of the gut microbiome was annotated according to the Kyoto Encyclopedia of Genes and Genomes database (KEGG database).

## 3. Results

### 3.1. Weight

**Figure 1:**
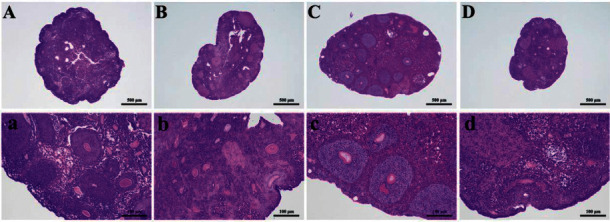
The pathological examination of ovaries in mice of each group: (a) and (b) mean control group; (c) and (d) mean POF group; (e) and (f) mean EA group; and (g) and (h) mean EN group.

All mice were weighed after treatment for 3 weeks. Although the weight of the EA group showed a recovery trend, and besides, a significant increase could also be observed than the POF group, the weight of the EA group was still slightly lower than the control group. Compared with the POF group, the weight of the EN group had no significant difference (Figure 1(c)).

### 3.2. Observation of Estrous Cycle

Vaginal exfoliated cell examination was applied to confirm the estrous cycle in mice. In the control group and EA group, nucleated epithelial cells, cornified epithelial cells, and leukocytes were observed alternately. There was long-term existence of nucleated epithelial cells and leukocytes, but cornified epithelial cells were not observed in the POF group and EN group. These results indicated that POF mice were established successfully (Figure S2).

### 3.3. Histopathology Examination

As shown in Figure 1, the antral follicles and corpus luteum could be observed in the control group. On the contrary, the number of atresia follicles increased in the POF group, which indicated the POF model was established successfully. There were more intact follicles in the EA group than in the POF group. Moreover, atresia follicles were decreased in the EA group. Although there were a few antral follicles in the EN group, atresia follicles and the structure of vacuoles could still be observed (Figure 1).

### 3.4. ELISA Examination

**Figure 2:**
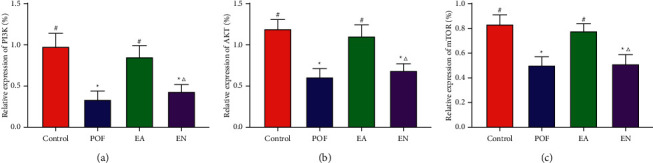
The relative expressions of PI3K, AKT, and mTOR in the ovary. ^*∗*^ means significantly different from the control group; ^#^ means significantly different from the POF group; and ^△^ means significantly different from the EA group.

Compared with the POF group, the levels of FSH and LH were significantly decreased, while the levels of E_2_, AMH, and *β*-glucuronidase were increased after EA. There was no significant difference in the levels of FSH, LH, AMH, and *β*-glucuronidase between the EN group and POF group. However, the level of E_2_ in the EN group was significantly higher than that in the POF group (Figure 2).

### 3.5. QPCR Examination

**Figure 3:**
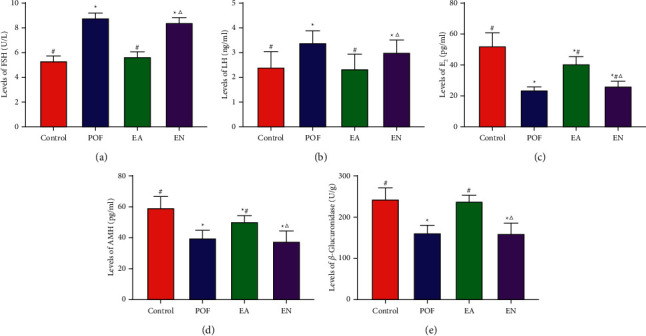
The levels of serum (a) FSH, (b) LH, (c) E_2_, (d) AMH, and (e) *β*-glucuronidase in different groups. ^*∗*^ means significantly different from the control group; ^#^ means significantly different from the POF group; and ^△^ means significantly different from the EA group.

The results showed that the relative expressions of PI3K, AKT, and mTOR in the POF group were lower than those in the control group. Obviously, there were no significant differences in the relative expression of PI3K, AKT, and mTOR between the EA group and control group. In addition, the expressions of PI3K, AKT, and mTOR in the EA group were significantly increased compared with those in the EN group (Figure 3).

### 3.6. Relative Content of Gut Microbiome

**Figure 4:**
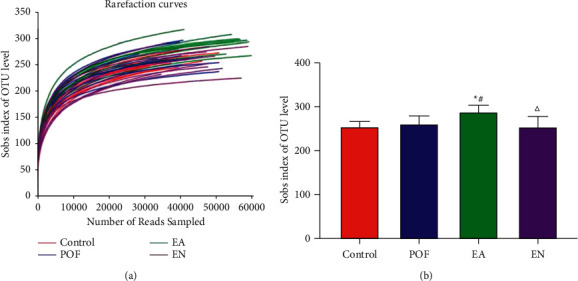
Sobs index and rarefaction curves of the gut microbiome at OTU level. ^*∗*^ means significantly different from the control group; ^#^ means significantly different from the POF group; and ^△^ means significantly different from the EA group.

The dual terminal sequence data were obtained by the MiSeq sequencing platform. Nonrepetitive sequences of OTUs were clustered according to more than 97% similarity. The rarefaction curves were constructed according to the Sobs index of each sample at different sequencing depths. With the increase of sequencing depth, the dilution curve tended to be flat. All in all, the sequencing depths of all samples were reasonable (Figure 4(a)).

In this study, there were no significant differences in the Sobs index of OTU level among the control group, POF group, and EN group. However, the Sobs index of OTU level in the EA group was significantly higher than that in the POF group (Figure 4(b)).

### 3.7. Diversity of Gut Microbiome

**Figure 5:**
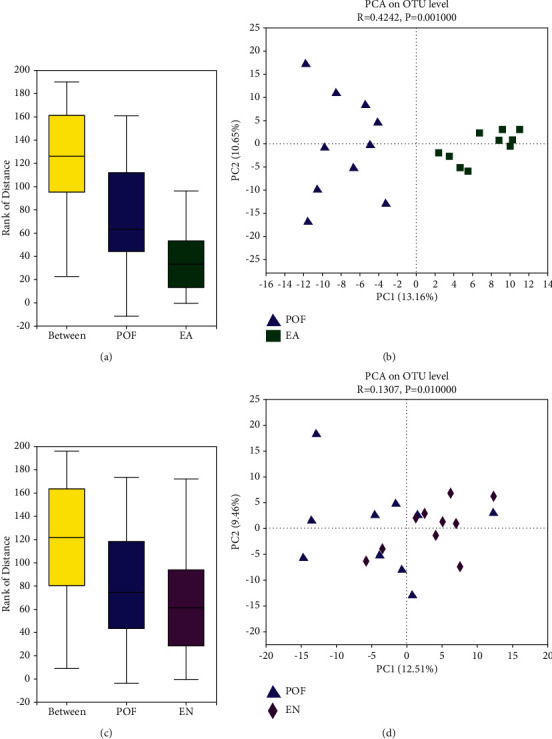
ANOSIM and PCA between the POF group and EA group ((a) and (b)) and between the POF group and EN group ((c) and (d)).

ANOSIM analysis and PCA indicated that the diversity of the gut microbiome in each group was significantly different (Figures S3-S6). As shown in the heatmap, the abundance and diversity of the gut microbiome were different in each group (Figure S7). PCA showed that the gut microbiome of the POF group and EA group had obvious dispersion, and there was a significant difference between the POF group and the EA group. Similarly, although the PCA showed that the diversity of gut microbiome in the POF group and EN group had poor dispersion, there was a significant difference between the POF group and EN group (Figure 5).

### 3.8. Species Changes of Gut Microbiome

**Figure 6:**
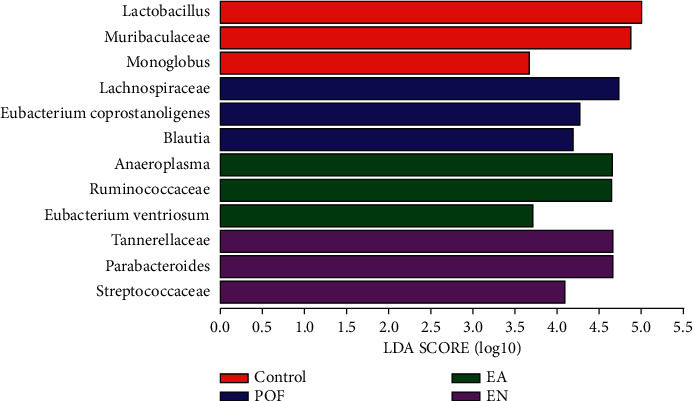
The histogram of LDA value distribution and the three dominant microbiomes with the highest relative expression were selected in each group.

To screen the different species of the microbiome in each group, LDA was applied and the top three most diverse and highest expression dominant microbiomes in each group were selected. According to the results, the dominant microbiomes of the control group were *Lactobacillus*, *Muribaculaceae*, and *Monoglobus*. Meanwhile, the dominant microbiomes in the POF group were *Lachnospiraceae*, *Eubacterium coprostanoligenes*, and *Blautia*. Then, *Anaeroplasma*, *Ruminococcaceae*, and *Eubacterium ventriosum* were the dominant microbiomes of the EA group. In addition, the dominant microbiomes of the EN group were *Tannerellaceae*, *Parabacteroides*, and *Streptococcaceae* (Figure 6).

To compare the relative abundance of the gut microbiome, the three highest expression dominant microbiomes in the control group were compared with other groups. The relative abundance of *Lactobacillus* in the EA group was significantly higher than that in the POF group and EN group. Then, the relative abundance of *Muribaculaceae* in the EA group was significantly lower than that in the control group. However, there was no significant difference in the relative abundance of *Muribaculaceae* between the EA group and POF group. Furthermore, the relative abundance of *Monoglobus* in the control group was significantly higher than that in other groups, but there was no significant difference between the POF group and EA group (Figure S8).

The *Firmicutes*/*Bacteroidetes* (F/B) ratio was considered to be an important indicator of gut microbiome homeostasis [11]. Although the F/B ratio in the EA group was significantly higher than that in the control group, the ratio in the EA group was significantly lower than that in the POF group. (Figure S9). Besides, the F/B ratio was positively correlated with the level of FSH and LH. The F/B ratio was negatively correlated with the level of E_2_, AMH, PI3K, Akt, mTOR, and *β*-glucuronidase (Figure S10).

According to the correlation analysis between clinical factors and gut microbiome, the dominant microbiomes in the control group and the EA group were positively correlated with E_2_, AMH, and *β*-glucuronidase. The dominant microbiomes in the POF group and the EN group were positively correlated with FSH and LH (Figure S11).

### 3.9. Functionality of Gut Microbiome

**Figure 7:**
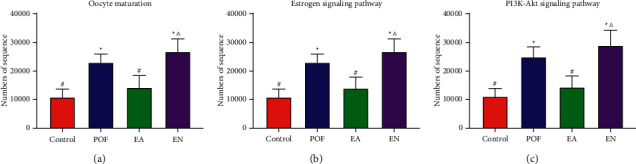
The KEGG database was applied to compare the levels of gut microbiome, related to estrogen signaling pathway, oocyte maturation, and PI3K-Akt signaling pathway in each group. ^*∗*^ means significantly different from the control group; ^#^ means significantly different from the POF group; and ^△^ means significantly different from the EA group.

According to the KEGG database, the estrogen signaling pathway, oocyte maturation, and PI3K-AKT signaling pathway were selected, which were the most related to POF, and the number of sequences was calculated. The results showed that the numbers of sequences associated with estrogen signaling, oocyte maturation, and PI3K-AKT signaling pathway were increased in the POF group. Meanwhile, the number of sequences in the EA group was lower than that of the POF group (Figure 7).

## 4. Discussion

Patients with POF usually have symptoms such as amenorrhea for more than four months, hot flashes, night sweats, low libido, and infertility [2]. Cisplatin is one of the most commonly used drugs in chemotherapy and is considered to activate PTEN through the PI3K-AKT signaling pathway, which might potentially lead to follicular atresia [12]. According to the theory of traditional Chinese medicine, POF is caused by the dysfunction of the kidney, liver, and spleen. The meridians that relate to the kidney, liver, and spleen all pass through the “SP6”. Therefore, Chinese medicine believes that ovarian function can be improved by “SP6”. Moreover, “CV4” is located at the Ren-meridian, which is considered to regulate menstruation and improve ovarian function [13]. As the above results, we found that EA could repair the injured ovary and restore the estrous cycle in cisplatin-induced POF mice in this study. On the contrary, EN had little effect on the regulation of ovarian pathological morphology and estrus cycle.

FSH, LH, E_2_, and AMH are the important sex hormones that can reflect ovarian function [2]. The decrease of AMH level indicates premature depletion of primordial follicles [14]. Follicular atresia causes the decrease of granulosa cells in follicles when POF occurs [15]. The long-term maintenance of low serum E_2_ levels will break the negative feedback mechanism between the ovary and pituitary and lead to increased levels of FSH and LH [16]. According to the results, it was found that EA could effectively restore the levels of FSH, LH, E_2_, and AMH in POF mice. However, EN did not restore the levels of sex hormones. These results might be related to EA increased the number of antral follicles, which showed that acupoints could improve ovarian function more effectively than nonacupoints.

The PI3K-AKT signaling pathway is involved in the process of oocyte growth, primordial follicle development, and granulosa cell proliferation [17]. FSH can combine with the receptor on the membrane of ovarian granulosa cells to activate the PI3K-AKT pathway to promote the maturation of ovarian granulosa cells [18]. Besides, E_2_ can combine with the estrogen receptor-*α* (ER-*α*) and estrogen receptor-*β* (ER-*β*) in the ovary [19]. ER-*α* combines with the subunit p85 in PI3K to activate AKT [20, 21]. Meanwhile, mTOR is activated by AKT, which leads to the proliferation and development of follicles [22]. In this study, we found that the relative expressions of PI3K, AKT, and mTOR were reversed to normal in POF mice by EA. The result indicated that the PI3K-AKT signaling pathway could be activated by electroacupuncture at acupoints to promote the proliferation of ovarian cells. The findings were consistent with the results in previous research of other subjects.

There are a large number of microbiomes in the human intestine, including probiotics, pathogenic bacteria, and conditioned pathogens [23]. The gut microbiome is considered to be closely related to human health [24]. Growing evidence shows that the disorder of the gut microbiome can lead to ovarian diseases, endocrine diseases, and cardiovascular diseases [25, 26]. It had been reported that the disorder of the gut microbiome would break the barrier of intestinal mucosa [27]. Pathogenic bacteria and their metabolites reached the ovary through the circulation of blood to reduce ovarian function [28]. A previous study showed that the diversity and abundance of dominant microbiomes in patients with premature ovarian insufficiency (POI) were altered. Meanwhile, the alteration of the gut microbiome was closely related to FSH, E_2_, LH, and AMH in patients with POI [29].

On the other hand, the enterohepatic circulation of estrogen is an important way to maintain the stability level of estrogen [30]. Serum free estrogen is converted into conjugated estrogen by the liver. Then, the conjugated estrogen is excreted into the gut with the bile [31]. *β*-glucuronidase is considered to be one of the metabolites of the gut microbiome. Conjugated estrogen is converted into free estrogen by *β*-glucuronidase [32]. Free estrogen enters the circulatory system of blood through intestinal resorption to promote the growth of follicles and relieve the symptoms of POF.

In the control group, the three highest relative expression dominant microbiomes were *Lactobacillus*, *Muribaculaceae*, and *Monoglobus*. *Lactobacillus* has an important role in maintaining human health and is considered one of the most important probiotics in the gut [33]. *Lactobacillus* can inhibit the growth and reproduction of pathogenic bacteria [34]. The level of estrogen is increased by transplantation of *Lactobacillus* in the gut to improve the ovarian function and estrous cycle of PCOS [35]. *Muribaculaceae* is widely distributed in the gut of mice. *Muribaculaceae* can reduce the colonization of *Clostridium difficile* in the intestine [36]. Meanwhile, *Muribaculaceae* can degrade carbohydrates. Moreover, *Monoglobus* can degrade pectin and maintain intestinal health [37]. According to the above results, the three highest relative expression dominant microbiomes were probiotics in the control group, which were important to maintain the healthy physiological function of the intestine.

*Lachnospiraceae*, *Eubacterium coprostanoligenes*, and *Blautia* were the dominant microbiomes in the POF group. *Lachnospiraceae* is considered to protect the intestinal mucosa [38]. On the contrary, *Lachnospiraceae* can damage the pathway of glucose metabolism and promote the process of inflammation [39]. Inflammation, aging, and autoimmune response are closely related to POF. Ovarian inflammation can be indirectly affected by *Lachnospiraceae* and is associated with TNF-*α* in direct proportion [40]. Therefore, *Lachnospiraceae* is considered as a conditional pathogen. *Eubacterium coprostanogenes* is regarded as pathogenic bacteria. Besides, *Eubacterium coprostanogenes* can reduce the intestinal absorption of cholesterol [41, 42]. *Blautia* can prevent inflammation and promote the production of short-chain fatty acids (SCFAs) to maintain intestinal homeostasis, so it is considered to be a potential probiotic [43, 44]. Whether the existence of beneficial bacteria in the first three dominant bacteria of POF mice is related to the self-healing function of mice still needs to be further researched.

In the EA group, *Anaeroplasma*, *Ruminococcaceae*, and *Eubacterium ventriosum* were the three microbiomes with the highest relative expression. *Anaeroplasma* is an anti-inflammatory agent. In addition, *Anaeroplasma* can maintain the immune homeostasis in intestinal mucosa [45]. Similarly, *Ruminococcaceae* and *Eubacterium ventriosum* have the anti-inflammatory function, and both are the common beneficial bacteria in the gut [46, 47]. As a member of SCFA producer, *Ruminococcaceae* is considered to maintain gut immune homeostasis. The levels of estrogen could be increased by *Ruminococcaceae*, which was reported in a cross-sectional study [48]. All the first three dominant microbiomes in POF mice were probiotics after EA. The result indicated that EA might increase the abundance and diversity of probiotics.

The three highest relative expression dominant microbiomes in the EN group were *Tannerellaceae*, *Parabacteroides*, and *Streptococcaceae*. *Tannerellaceae* promotes the occurrence of intestinal inflammation [49]. *Parabacteroides* are considered to be beneficial bacteria [50]. Moreover, *Parabacteroides* can alleviate liver injury and reduce the expression of inflammatory genes in the liver [51]. The increase of *Parabacteroides* in the gut is closely related to the improvement of ovarian function [52]. *Streptococcaceae* is a conditioned pathogen, which is a common microbiome in the human oral cavity, skin, intestinal, and upper respiratory tract [53]. The increase of *Streptococcaceae* is inversely proportional to E_2_. Moreover, the alteration of the gut microbiome can affect metabolic homeostasis, which is mediated by E_2_ [54]. Although the content of probiotics could be increased by EN in this study, the dominant bacteria were pathogenic bacteria.

EA could significantly reduce the F/B ratio, which indicated that EA had a good effect on improving the structure of the gut microbiome. The abundance and diversity of the gut microbiome in POF mice could be regulated by EA.

Oocyte maturation is the initial stage of follicle development. The proliferation of granulosa cells can be promoted by PI3K-Akt signaling pathway activation in follicles. The estrogen signaling pathway existed in the cell nucleus and cell membrane, which can activate the PI3K-Akt signaling pathway in the ovary. According to the result of KEGG pathway analysis, the gut microbiome, which was related to the estrogen signaling pathway, oocyte nutrition, and PI3K-AKT signaling pathway, was regulated by EA. The finding indicated that EA might play a role in the treatment of POF by regulating the gut microbiome.

The disease from POF would become an increasing public health burden. Regulating the gut microbiome by EA to impact ovarian function provides an exciting future therapeutic. This study provided a preliminary verification for revealing the mechanism of EA in the treatment of POF. In the future, antibiotic cocktail mice would be selected to further research the therapeutic mechanism of EA on POF by faecal microbial transplantation and macro gene sequencing.

## 5. Conclusions

EA could restore ovarian pathological morphology and the estrous cycle of mice with POF. In the meantime, sex hormones and the PI3K-AKT signaling pathway in mice with POF were regulated by EA. Furthermore, we speculated that the estrogen level might be restored by EA through regulating the abundance and the diversity of the gut microbiome in POF mice. Estrogen combined with the estrogen receptor on follicular granulosa cells could activate the PI3K-AKT signaling pathway and could promote the growth and development of follicles.

## Data Availability

The original data used to support the funding of this study were supplied by Min Chen under license. Requests for access to these data should be made to mchen@must.edu.mo.

## Supplementary Materials

Supplementary Materials
*Supplementary Figure S1*. The selected points and weight of mice: (A and B) acupoints and nonacupoints and (C) the weight of mice in each group. *Supplementary Figure S2*. The vaginal exfoliated cells reflected different estrous cycle in each group. *Supplementary Figure S3*. (A) ANOSIM and (B) PCA in the control group and POF group. *Supplementary Figure S4*. (A) ANOSIM and (B) PCA in the control group and EA group. *Supplementary Figure S5*. (A) ANOSIM and (B) PCA in the control group and EN group. *Supplementary Figure S6*. (A) ANOSIM and (B) PCA in the EA group and EN group. *Supplementary Figure S7*. The heatmap of the most differentially expressed microbiomes in different groups. *Supplementary Figure S8*. The relative expression levels of the first three dominant bacteria in the control group were compared with those in the other groups. *Supplementary Figure S9*. The *Firmicutes/Bacteroidetes* (F/B) ratios of all the groups. *Supplementary Figure S10*. The correlation analysis (Pearson's correlation coefficient) between the blood samples, ovary samples, and *Firmicutes/Bacteroidetes* (F/B) ratio. *Supplementary Figure S11*. The correlation analysis between gut microbiome and clinical factors.
