# Supplementary material for: Effects of Electroacupuncture on the Gut Microbiome in Cisplatin-Induced Premature Ovarian Failure Mice
Source: Evid Based Complement Alternat Med. 2022 Mar 14;2022:9352833. doi: 10.1155/2022/9352833 (PMC8938064; doi:10.1155/2022/9352833)
Supplement: Supplementary Materials — Supplementary Figure S1. The selected points and weight of mice: (A and B) acupoints and nonacupoints and (C) the weight of mice in each group. Supplementary Figure S2. The vaginal exfoliated cells reflected different estrous cycle in each group. Supplementary Figure S3. (A) ANOSIM and (B) PCA in the control group and POF group. Supplementary Figure S4. (A) ANOSIM and (B) PCA in the control group and EA group. Supplementary Figure S5. (A) ANOSIM and (B) PCA in the control group and EN group. Supplementary Figure S6. (A) ANOSIM and (B) PCA in the EA group and EN group. Supplementary Figure S7. The heatmap of the most differentially expressed microbiomes in different groups. Supplementary Figure S8. The relative expression levels of the first three dominant bacteria in the control group were compared with those in the other groups. Supplementary Figure S9. The Firmicutes/Bacteroidetes (F/B) ratios of all the groups. Supplementary Figure S10. The correlation analysis (Pearson's correlation coefficient) between the blood samples, ovary samples, and Firmicutes/Bacteroidetes (F/B) ratio. Supplementary Figure S11. The correlation analysis between gut microbiome and clinical factors. [file 9352833.f1.docx]

# [Evidence-Based Complementary and Alternative Medicine](https://www.hindawi.com/journals/ecam/)

# Effect of Electro-acupuncture on Gut Microbiome in Cisplatin-Induced Premature Ovarian Failure Mice

Qi-da He, ^1,2,#^ Jing-jing Guo, ^3,#^ Qi Zhang, ^3^ Yuen-ming Yau, ^3^ Yue Yu, ^1,2^ Zheng-hong Zhong, ^1,2^ Zi-yan Tong,^1,2^ Zong-bao Yang , ^3*^ and Min Chen ^1,2*^

^1^ Faculty of Chinese Medicine, Macau University of Science and Technology, Macau 999078, China
^2^ State Key Laboratory of Quality Research in Chinese Medicines, Macau University of Science and Technology, Macau 999078, China

^3^ Department of Traditional Chinese Medicine, Xiamen University, Xiamen 361000, China

Correspondence should be addressed to Min Chen; [mchen@must.edu.mo](mailto:mchen@must.edu.mo); Zong-bao Yang; yangzb@xmu.edu.cn

^#^ These authors have contributed equally to this work and share first authorship. ^*^ These authors contributed equally to this work.


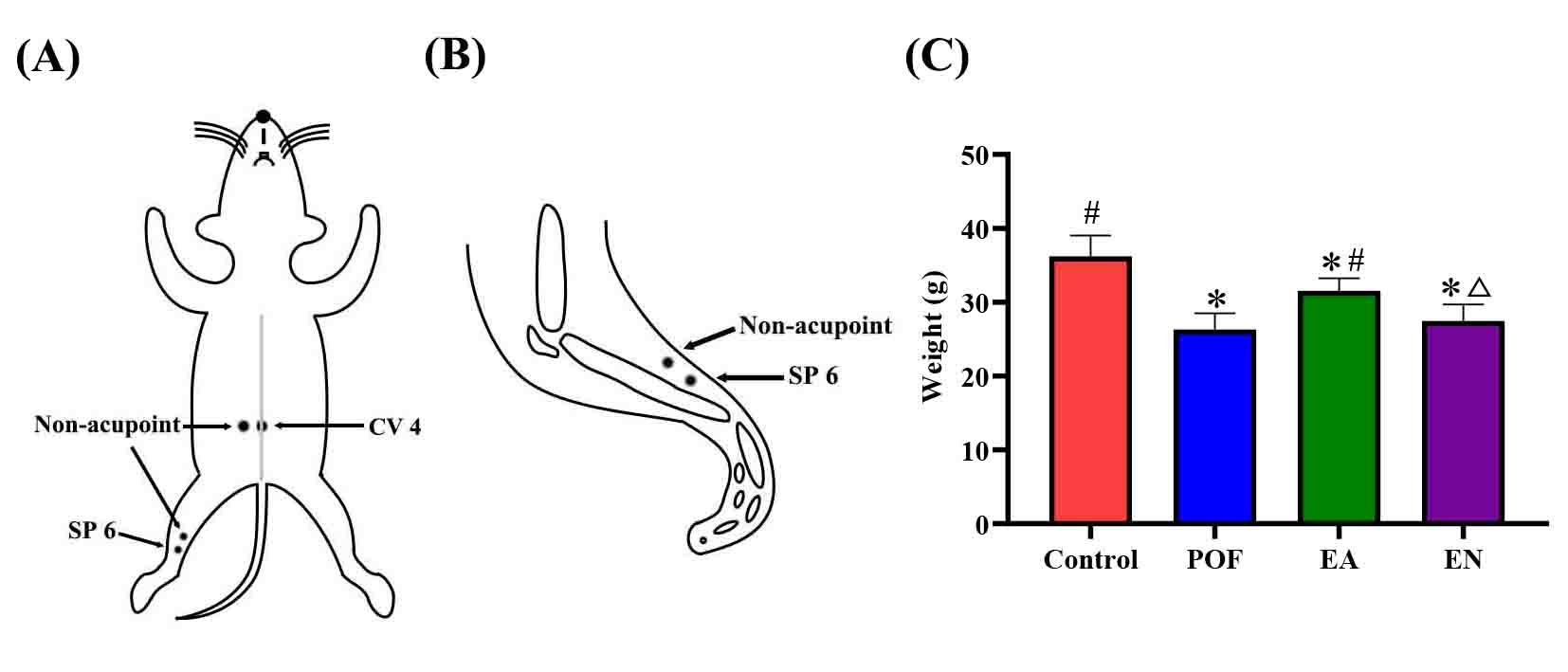


Figure S1: The selected points and weight of mice. A and B: acupoints and non-acupoints. C: the weight of mice in each group (*mean significant difference from the control group at *P*<0.05; # mean significant difference from the POF group at *P*<0.05; △mean significant difference from the EA group at *P*<0.05.).


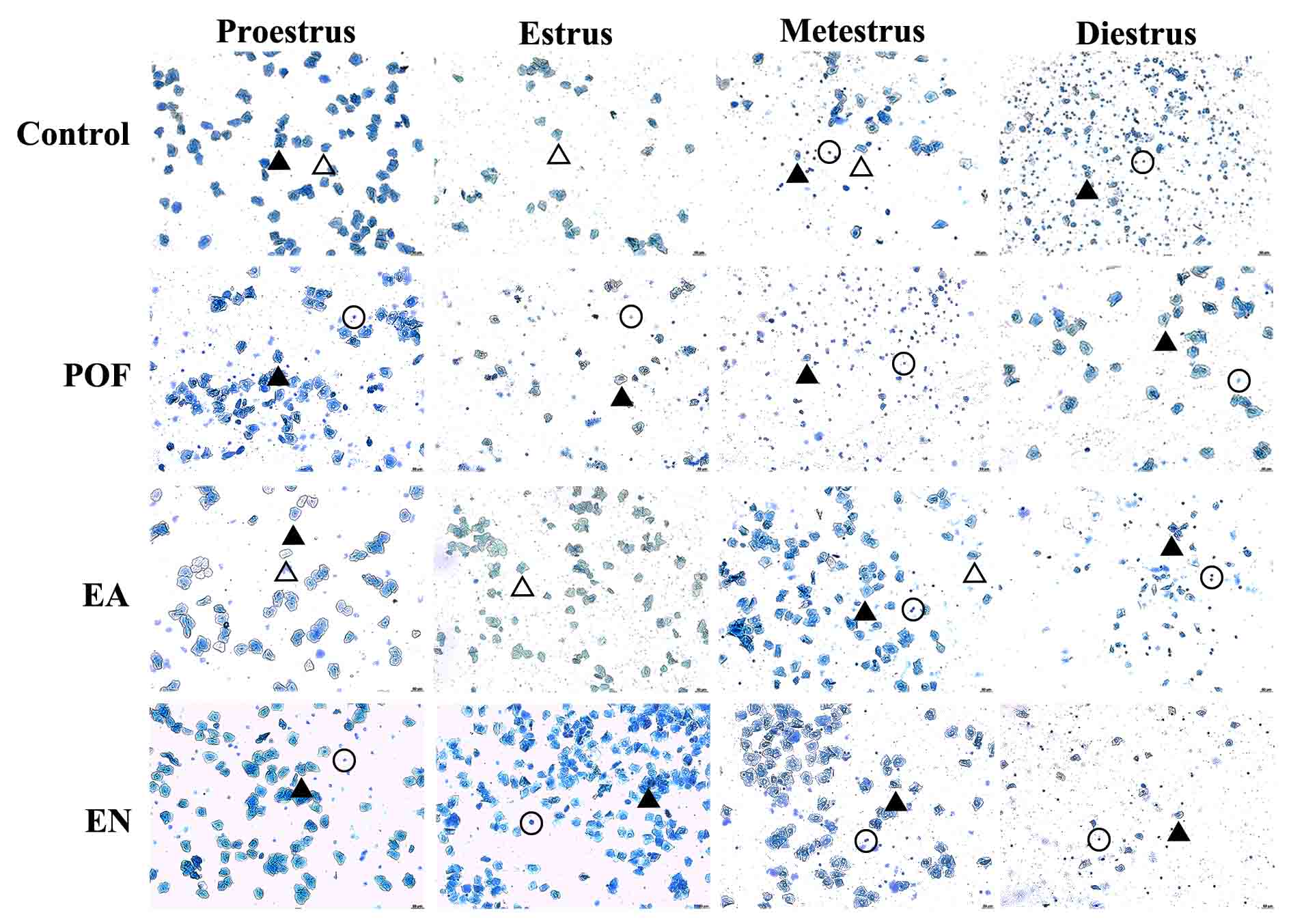


Figure S2: The vaginal exfoliated cells reflected different estrous cycle in each group. Nucleated epithelial (black triangle), cornified epithelial (blank triangle), and leukocytes (circle).


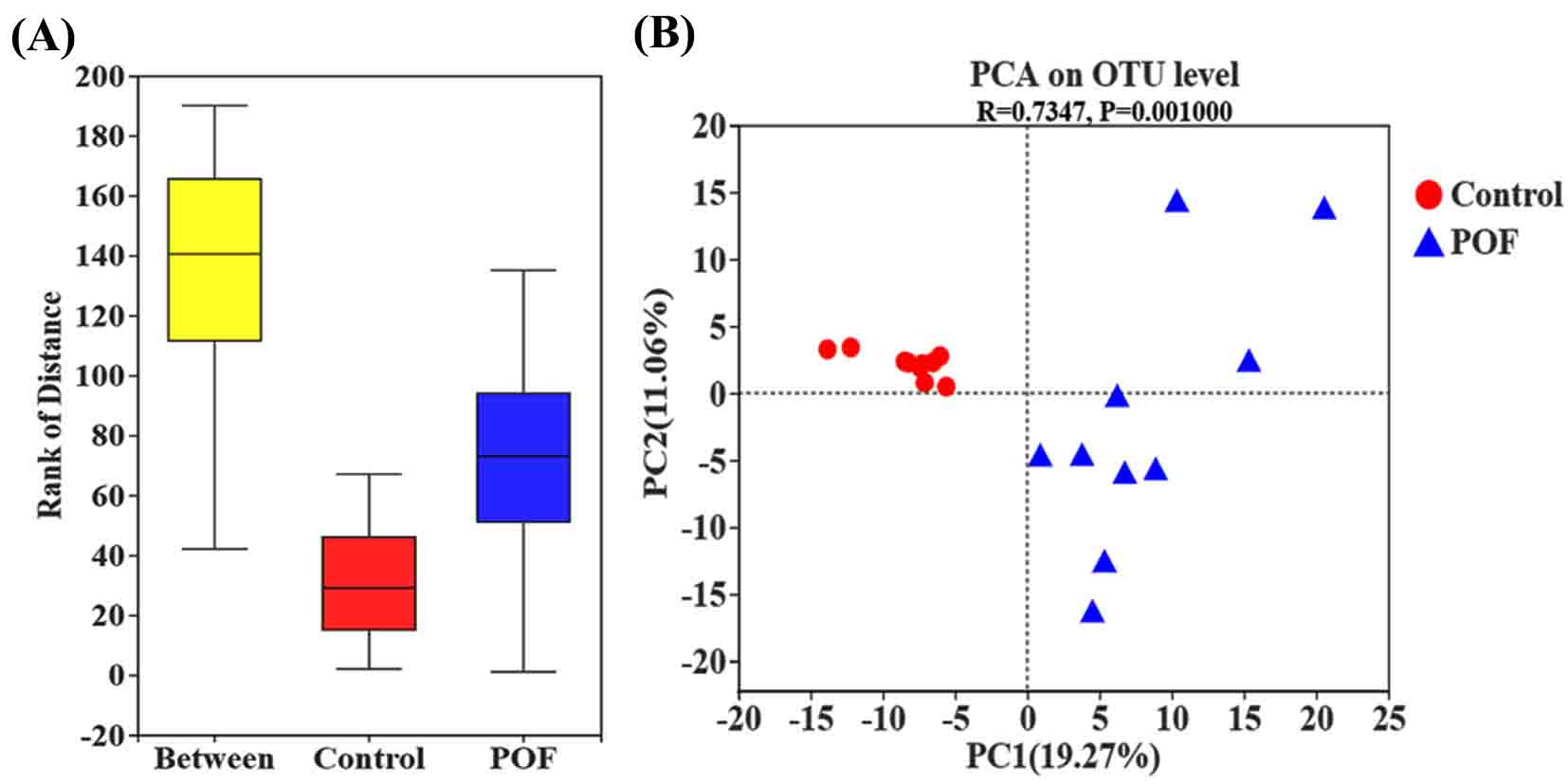
Figure S3: ANOSIM and PCA analysis in (A and B) the control group and POF group.


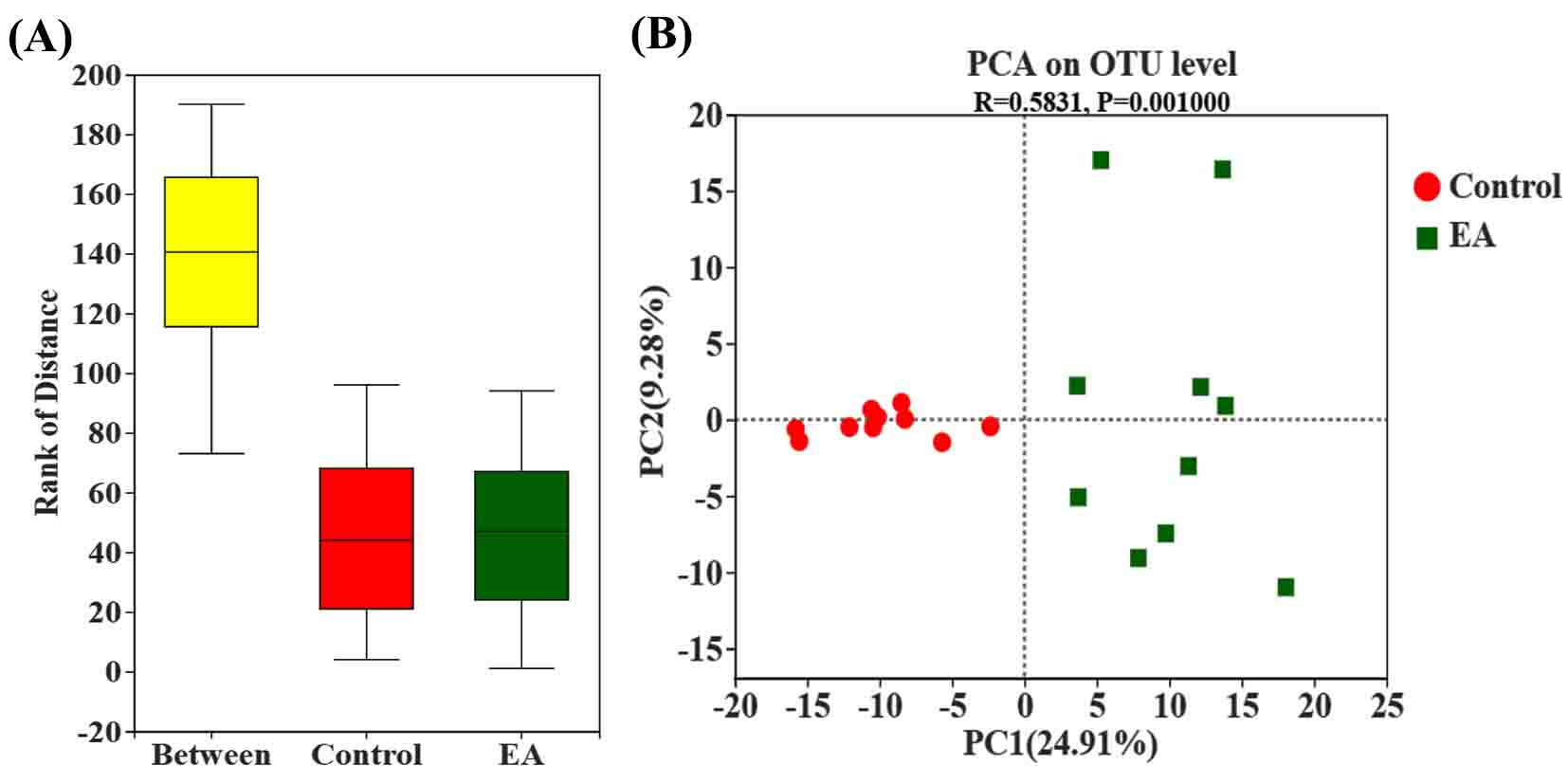


Figure S4: ANOSIM and PCA analysis in (A and B) the control group and EA group.


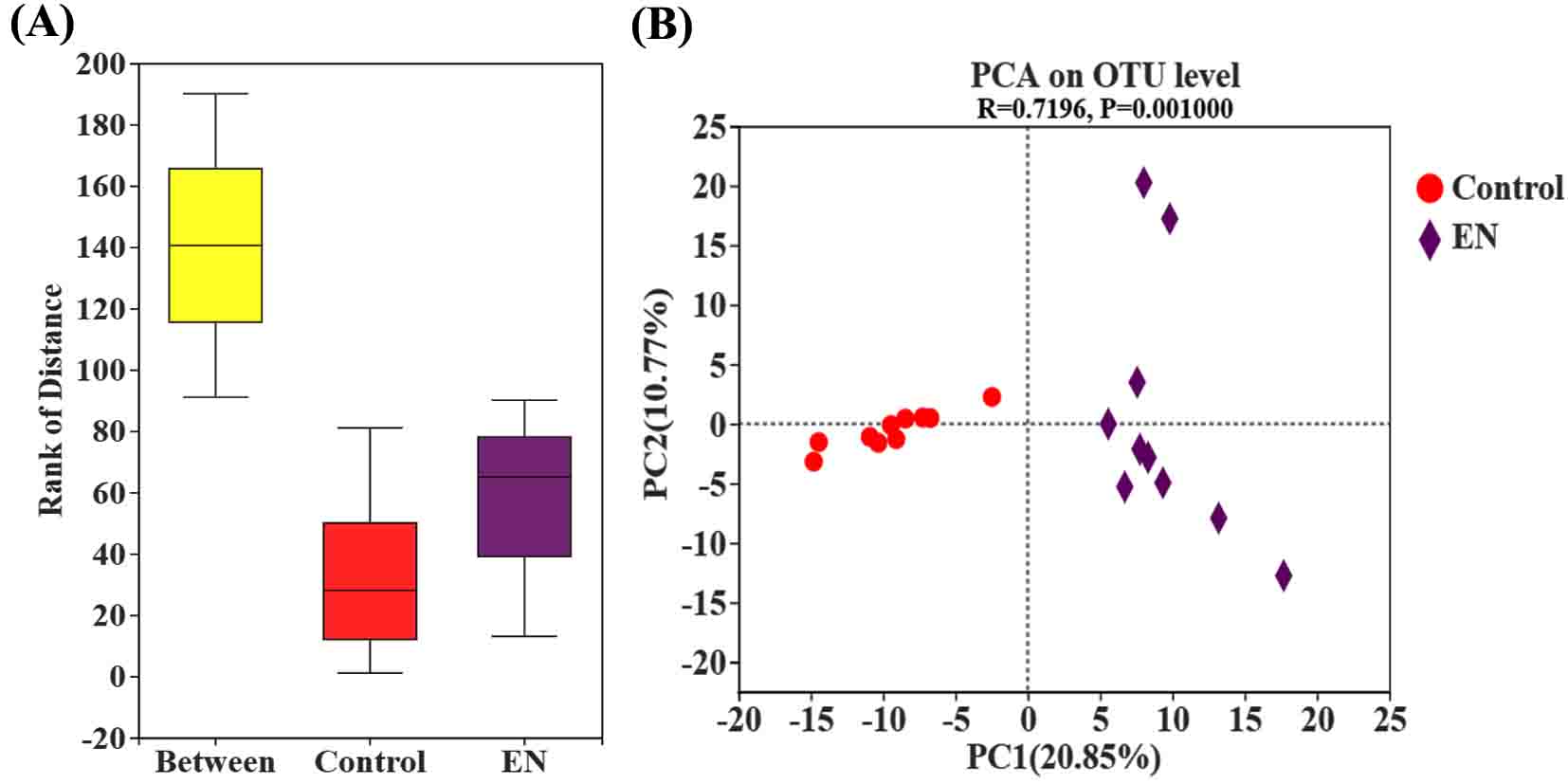


Figure S5: ANOSIM and PCA analysis in (A and B) the control group and EN group.


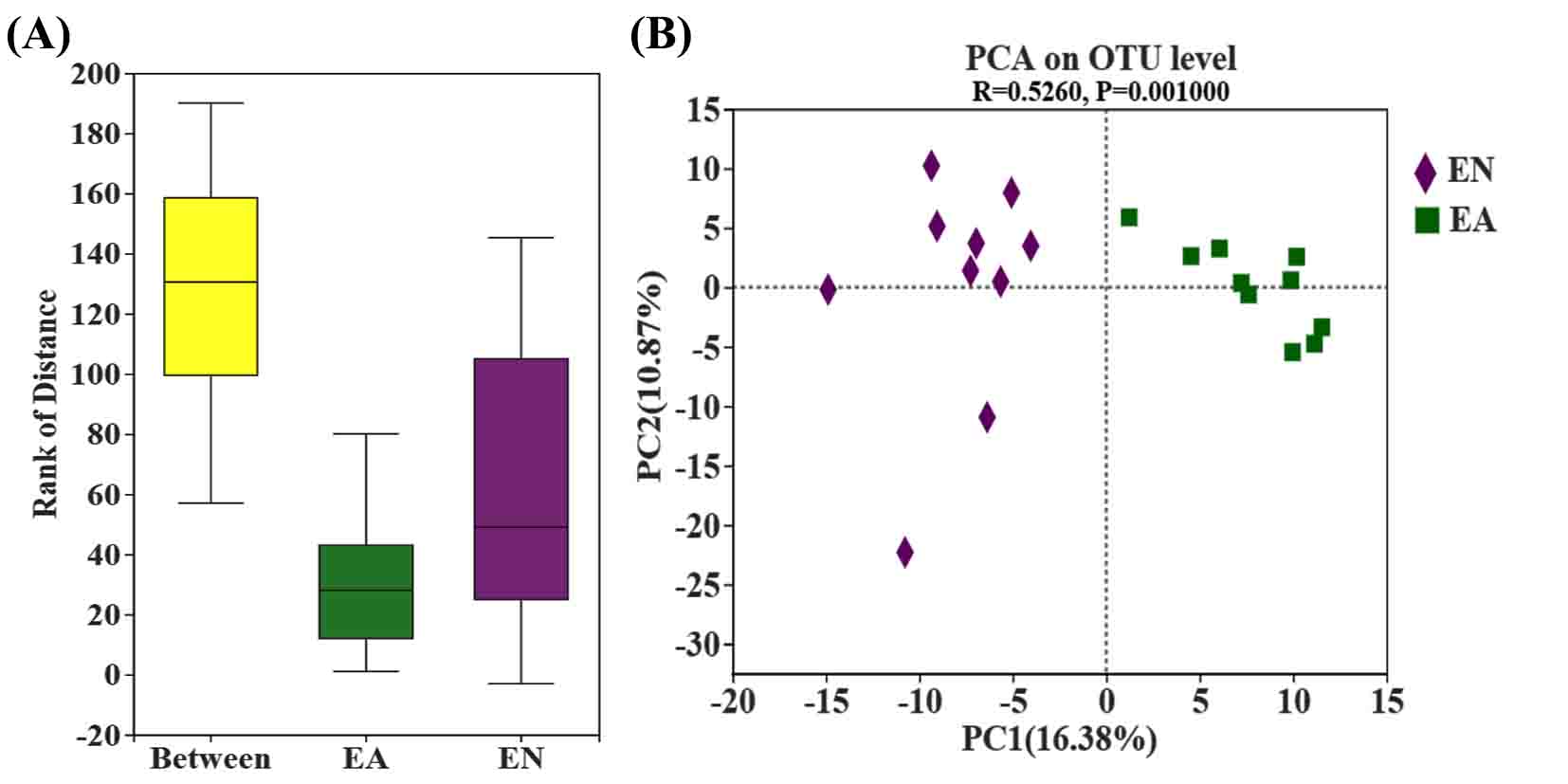


Figure S6: ANOSIM and PCA analysis in (A and B) the EA group and EN group.


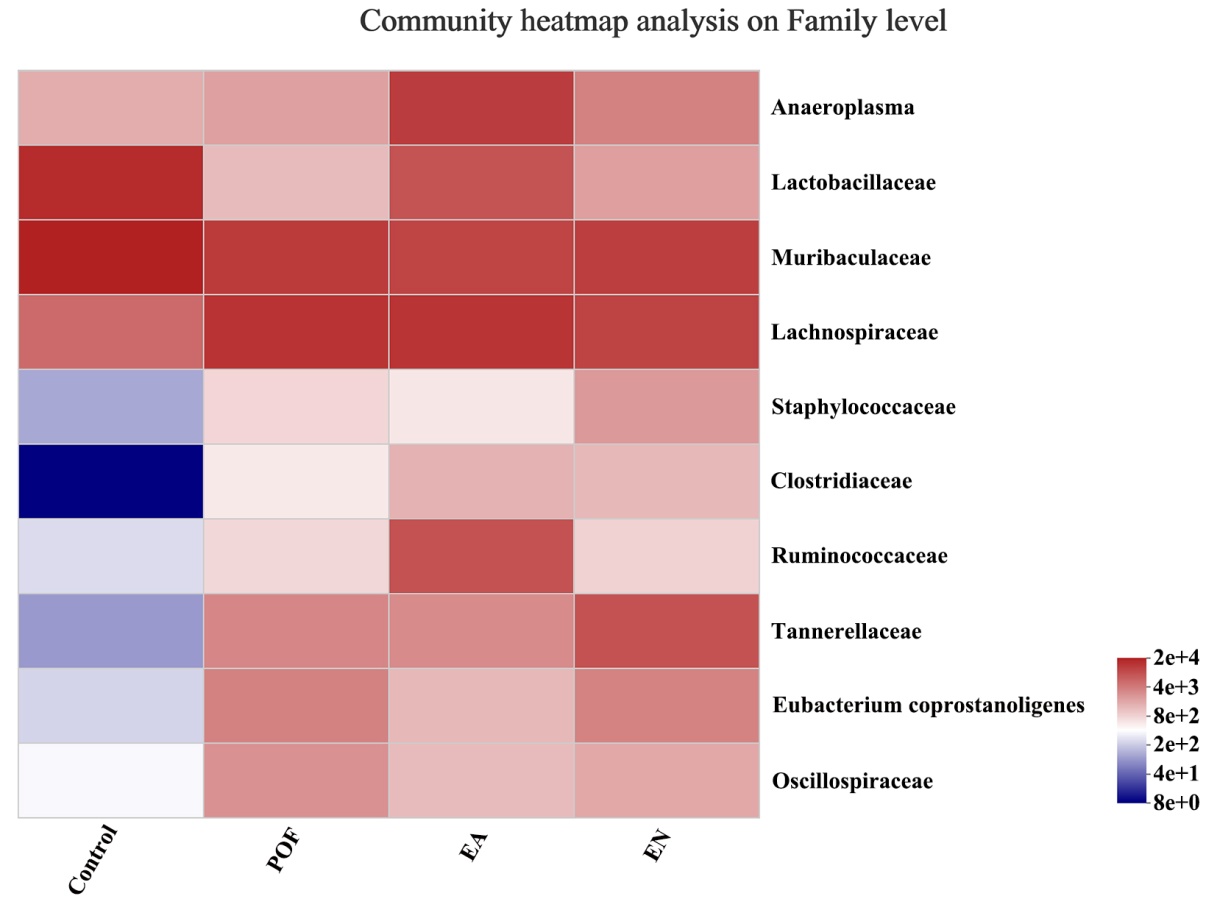


Figure S7. The heatmap of the most differentially expressed microbiomes in different groups.


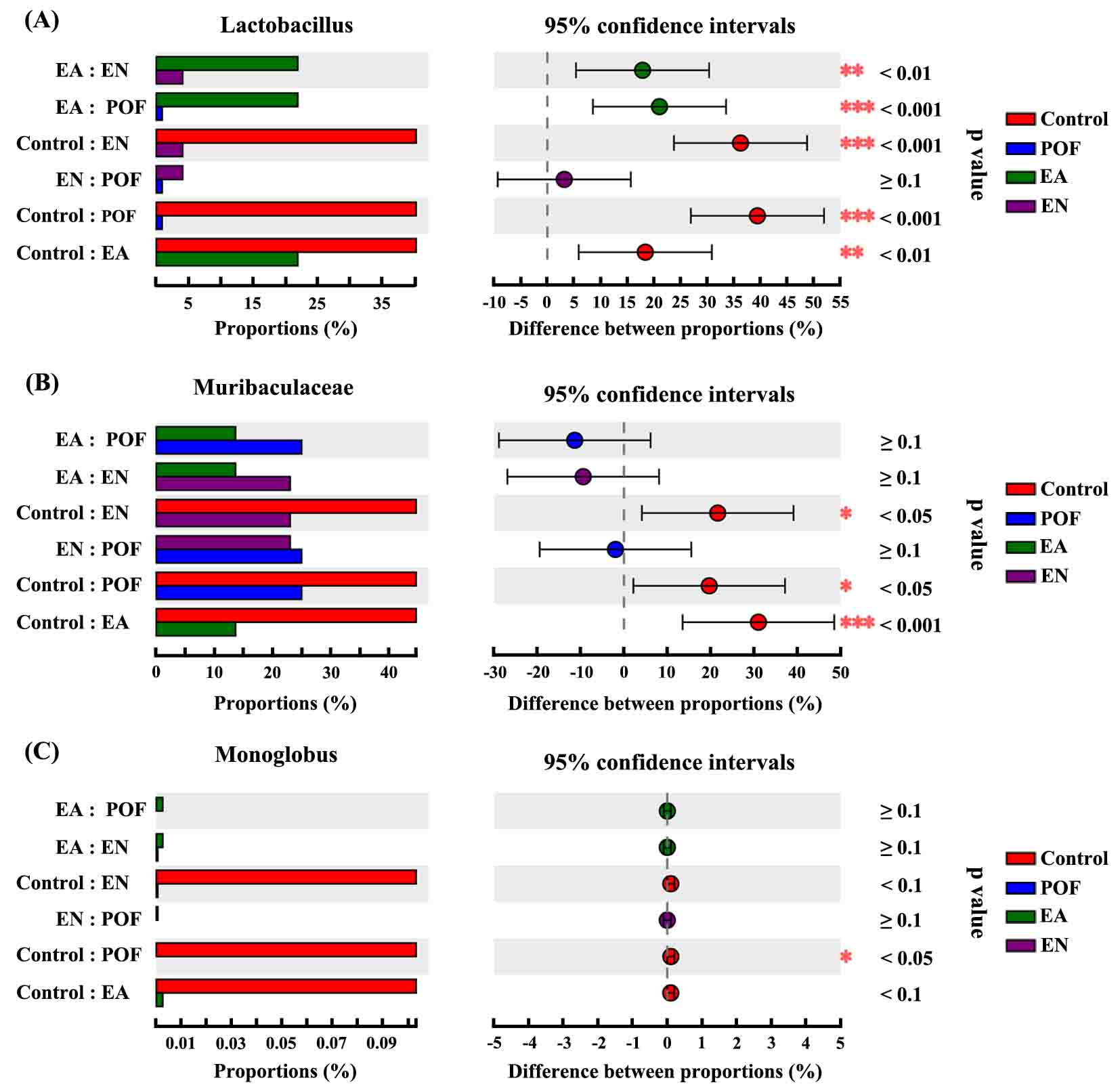


Figure S8: The relative expression levels of the first three dominant bacteria in control group were compared with those in the other groups.


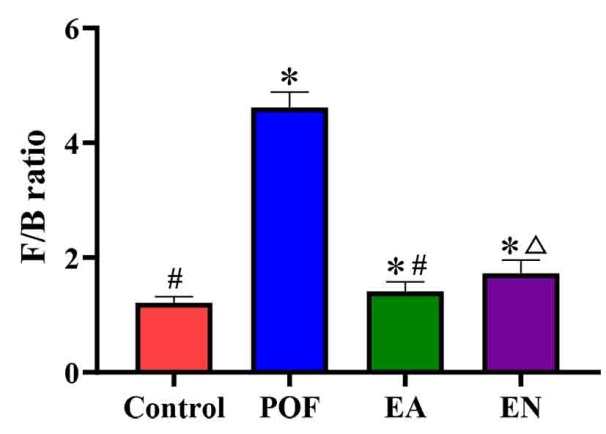


Figure S9: The *Firmicutes*/*Bacteroidetes* (F/B) ratios in groups. (*means significant difference from the control group; # means significant different from the POF group; △means significant different from the EA group.)


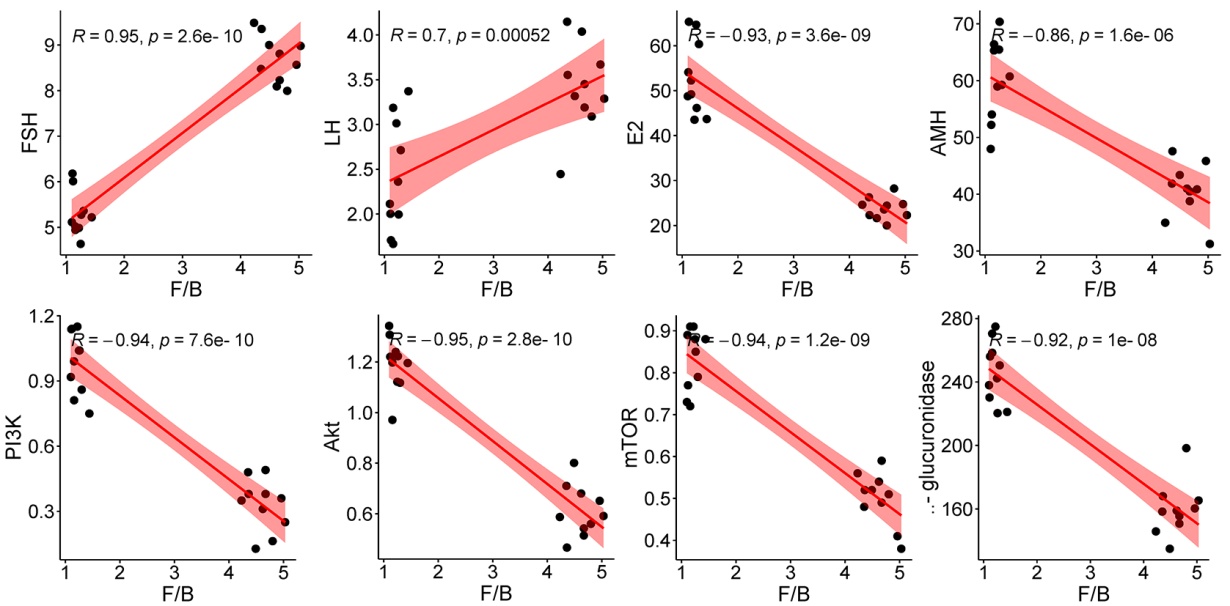


Figure S10. The correlation analysis (pearson correlation coefficient) between the blood samples, ovary samples and *Firmicutes/Bacteroidetes* (F/B) ratio.


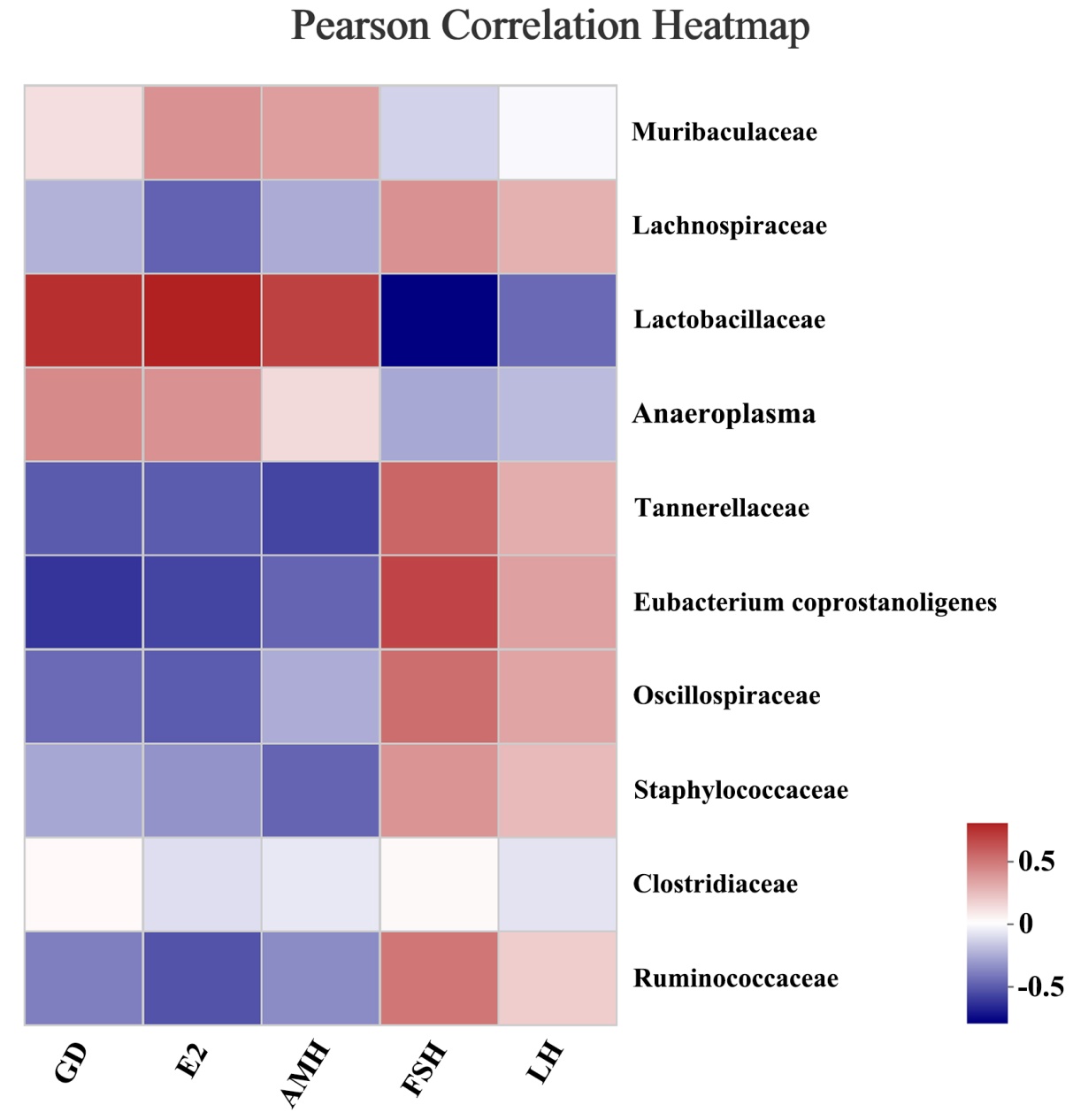


Figure S11. The correlation analysis between gut microbiome and clinical factors.
